# Pathotype determination of sorghum anthracnose (*Colletotrichum sublineola*) isolates from Ethiopia using sorghum differentials

**DOI:** 10.3389/fmicb.2024.1458450

**Published:** 2024-10-09

**Authors:** Moges Mekonen, Kassahun Tesfaye, Tesfaye Mengiste, Alemayehu Chala, Habte Nida, Tilahun Mekonnen, Kibrom B. Abreha, Mulatu Geleta

**Affiliations:** ^1^Chiro National Sorghum Research and Training Center, Ethiopian Institute of Agricultural Research, Addis Ababa, Ethiopia; ^2^Institute of Biotechnology, Addis Ababa University, Addis Ababa, Ethiopia; ^3^Bio and Emerging Technology Institute, Addis Ababa, Ethiopia; ^4^Department of Botany and Plant Pathology, Purdue University, West Lafayette, IN, United States; ^5^College of Agriculture, Hawassa University, Hawassa, Ethiopia; ^6^Department of Plant Breeding, Swedish University of Agricultural Sciences (SLU), Alnarp, Sweden

**Keywords:** anthracnose, *Colletotrichum sublineola*, disease resistance, genotypes, landraces, virulence spectrum

## Abstract

**Introduction:**

Sorghum anthracnose, caused by *Colletotrichum sublineola*, is the most destructive disease of sorghum, which causes up to 80% grain yield loss in susceptible varieties. The use of resistance varieties is an effective, durable, and eco-friendly strategy for anthracnose control. Knowledge of the phenotypic and genetic variation in *C. sublineola* is vital for designing appropriate anthracnose management strategies.

**Methods:**

The present study examined the morphology and virulence of 25 *C. sublineola* isolates recovered from various sorghum-producing regions of Ethiopia against 18 known sorghum anthracnose differentials, 6 Ethiopian sorghum landraces, and a variety of Bonsa.

**Results:**

Analysis of variance (ANOVA) revealed significant differences among sorghum genotypes, *C. sublineola* isolates, and their interactions. There was a significant difference between the isolates in virulence, with each isolate exhibiting virulence in 8–72% of the sorghum genotypes tested. Among the 25 tested isolates, the top four most virulent isolates were from Pawe, suggesting that this area is suitable for pathogen diversity studies and host plant resistance screening. The sorghum genotypes IS_18760, Brandes, and Bonsa showed resistance to all tested isolates. Consequently, they may provide potential sources of resistance genes for sorghum breeding programs to develop cultivars resistant to different *C. sublineola* pathotypes. However, the resistant check SC748-5 was susceptible to isolates NK73_F37, while another resistant check SC112-14 was susceptible to isolates PW123_F47 and PW122_F47. Cluster analysis grouped 22 isolates into seven clusters based on their morphological characters, whereas 24 pathotypes were identified among 25 isolates that were tested on 25 sorghum genotypes.

**Discussion:**

Hence, this study revealed high variation in *C. sublineola* in Ethiopia suggesting the need for broad-spectrum resistance to control the disease. Sorghum genotypes resistant to various *C. sublineola* isolates were identified in this study, which can be used in sorghum breeding programs aiming to develop resistant cultivars to anthracnose. Highly virulent *C. sublineola* isolates were also identified which could be used in sorghum germplasm resistance screening. The report is the first to show the existence of *C. sublineola* pathotypes in Ethiopia.

## Introduction

Sorghum (*Sorghum bicolor* (L.) Moench) is among the major cereal crops grown in several regions of the world due to its versatility and adaptability to diverse climatic conditions (Charles et al., 2009). The crop performs well at altitudes ranging from 500 to 1700 m above sea levels (masl) with seasonal rainfall of 300 mm and above. More than 500 million people in over 30 countries depend on sorghum, making it the world’s fifth most important crop for human consumption after rice, wheat, maize, and potatoes (Acquaah, 2012; Martin, 2016; Bunker et al., 2019; Ahn, 2020). It has diverse uses worldwide with all plant parts used for different purposes (Charles et al., 2009). The global production of sorghum increased from 55 million metric tons (MMT) in 2000 to 62.12 MMT in 2021 (FAOSTAT, 2023). Sorghum is one of Ethiopia’s major staple and strategic food security crops (Bezabeh, 2015) ranking third after teff and maize in area coverage and fourth after maize, teff, and wheat in total production (ESS, 2022). A total of 4.45 MMT of sorghum is estimated to be produced on 1.65 million hectares of land in the country (ESS, 2022). However, the productivity of sorghum in Ethiopia is at 2.6 tons per hectare (tha^−1^) (ESS, 2022), which is below the average grain yield in the United States, China, and the European Union, which is 4.4, 4.76, and 5.l tha^−1^, respectively (USDA, 2021).

Drought, diseases, low soil fertility (nutrient deficiencies), insects (stem borer and weevil), quelea birds, striga, and weeds have been recognized as major sorghum production constraints across the world (Charles et al., 2009; Girma et al., 2020). Of these, anthracnose caused by the fungus *Colletotrichum sublineola* is an important disease causing a substantial loss in grain, forage, and stover yields in the hot humid regions of the world (Thakur et al., 2007; Chala et al., 2010). Anthracnose severity of up to 87% was reported in Ethiopia (Tsedaley et al., 2016). Under maximum disease severity, grain loss could reach 86% in Brazil (Cota et al., 2017) and 80% in the USA (Prom et al., 2023).

Despite the economic significance of the disease in Ethiopia, information on sorghum *C. sublineola* pathotypes and virulence patterns is highly limited. Knowledge of the pathogen virulence variability is essential for deploying durable and effective management strategies. The identification and deployment of resistance genes into improved varieties susceptible to *C. sublineola* is an effective approach to managing sorghum anthracnose (Prom et al., 2012). Anthracnose resistance genes are believed to be widespread due to the high diversity of both the crop and the pathogen in their primary centers of origin, Ethiopia and Sudan (Thakur et al., 2007; Chala et al., 2010) including some genes that provide broad-spectrum resistance (Cuevas et al., 2019). Furthermore, previous studies have hinted at the potential existence of resistant sorghum landraces in Ethiopia that could be utilized for the development of sorghum cultivars resistant to anthracnose (Cuevas et al., 2019; Prom et al., 2022). Hence, investigating the distribution of *C. sublineola* pathotypes and identifying resistance sources could greatly help to address the challenge of sorghum production due to anthracnose in Ethiopia and beyond. This study is, therefore, primarily aimed at determining the virulence variability of 25 *C. sublineola* isolates collected from major sorghum growing areas in Ethiopia. Additionally, the study aimed at identifying sorghum genotypes resistant to anthracnose for use in sorghum breeding programs.

## Materials and methods

### Sample collection and isolation

**Figure 1:**
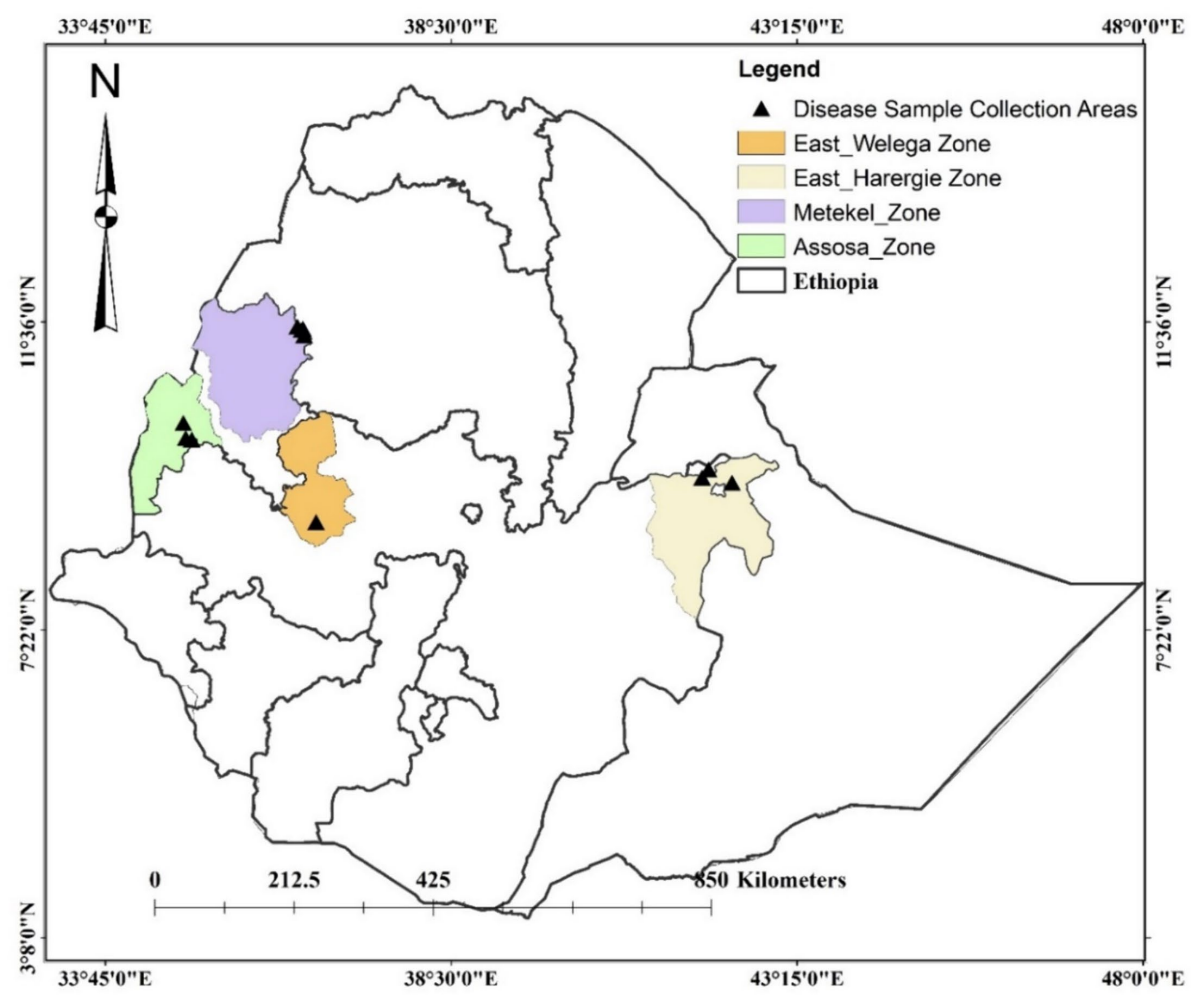
Map of Ethiopia showing *C. sublineola* isolate collection sites.

Sorghum leaf samples, with typical symptoms of anthracnose, were collected from Assosa, Haramaya, Nekamte, and Pawe districts in Ethiopia (Figure 1). These locations are categorized by hot and humid weather conditions, which are conducive to pathogen infection and proliferation. Assosa and Pawe districts are in the Assosa and Metekel zones, respectively, of the Benishangul-Gumuz Regional State. The sampling site in **Assosa** and Pawe had an altitude range of 1,426–1547.5 masl and 1,112–1,175 masl, respectively (Table 1). Assosa’s mean annual temperature and rainfall were 22.5°C and 1912.77 mm, respectively, whereas Pawe’s were 24.7°C and 1,254 mm. Haramaya and Nekamte districts are situated in East Hararge and East Wollega zones, respectively, of Oromia Regional State. The sampling sites in Haramaya had an altitude range of 2019–2088 masl, while the sampling site in Nekamte had an altitude of 1722 masl (Table 1). Harmaya’s mean annual temperature and rainfall were 20.8°C and 567.17 mm, respectively, while Nekamte’s were 21.44°C and 1306.16 mm.

**Table 1:** Geographic origin of 25 *C. sublineola* isolates used for pathotype analysis.

Isolates*	Administrative zone	Latitude (N)	Longitude (E)	Altitude (m)
AS100_F43	Assosa	10°0.02.647′	034°0.33.972′	1547.5
AS104_F43	Assosa	10°0.02.647′	034°0.33.972′	1547.5
AS105_F43	Assosa	10°0.02.647′	034°0.33.972′	1547.5
AS106_F43a	Assosa	10°0.02.647′	034°0.33.972′	1547.5
AS106_F43a	Assosa	10°0.02.647′	034°0.33.972′	1547.5
AS108_F43	Assosa	10°0.02.647′	034°0.33.972′	1547.5
AS110_F43	Assosa	10°0.02.647’	034°0.33.972’	1547.5
AS111_F43	Assosa	10°0.02.647’	034°0.33.972’	1547.5
AS89_F40	Assosa	09° 47.729’	034° 42.843’	1,426
AS90_F40	Assosa	09° 47.729’	034° 42.843’	1,426
AS74_F33*	Assosa	09° 56.261’	034° 39.731’	1,444
HU14_F02*	East Hararge	09° 23.284’	041° 55.099’	2060
HU22_F04	East Hararge	10° 22.092’	042° 55.134’	2088
HU28_F06	East Hararge	09° 24.623’	042° 02.003’	2019
HU34_F06	East Hararge	09° 24.623’	042° 02.003’	2019
NK73_F37	East Wollega	08° 42.534’	036° 27.690’	1722
PW117_F46	Metekel	11° 18.860’	036° 24.668’	1,112
PW122_F47	Metekel	11° 18.860’	036° 24.668’	1,112
PW123_F47	Metekel	11° 18.860’	036° 24.668’	1,112
PW125_F47	Metekel	11° 18.860’	036° 24.668’	1,112
PW74_F34	Metekel	11° 15.879’	036° 27.692’	1,162
PW83_F37	Metekel	11° 15.921’	036° 27.761’	1,163
PW90_F40	Metekel	11° 15.593’	036° 28.043’	1,175
PW84_F37	Metekel	11° 15.921’	036° 27.761’	1,163
PW85_F37	Metekel	11° 15.921’	036° 27.761’	1,163

Where “*” = were not morphologically characterized.

A total of 372 diseased leaf samples of sorghum were collected during the 2020 and 2022 cropping seasons from 142 sorghum fields. The sampled sorghum fields in a district were at least 5 km apart. Sample collection was made at crop growth stages (GS) from complete anthesis to the milking stage. Sampling was done at one to three random points within a farmer’s field. In the Assosa, Haramaya, and Pawe research stations, 10–15 samples were collected because of the plants’ high phenotypic diversity and their diverse disease symptoms. A paper bag labeled with a sample code was used to collect anthracnose symptomatic leaves of each sorghum genotype and transport them to the plant pathology laboratory of Melkassa Agricultural Research Center (MARC) of the Ethiopian Institute of Agricultural Research (EIAR) for analysis. Table 1 presents sample passport data, such as administrative regions, geographical coordinates, and altitudes. For pathogen isolation, infected leaf samples were surface sterilized with 1% sodium hypochlorite for 30 s followed by three times rinsing with sterilized distilled water. Sterilized leaves were allowed to dry in the laminar flow cabinet and transferred to 9 cm Petri dishes with potato dextrose agar (PDA; potato 200 g/L, dextrose 20 g/L, agar 15 g/L) (Sigma-Aldrich-70139) amended with 100 mg of chloramphenicol (Sigma-Aldrich-C0378) and incubated at 26°C for 5–7 days under 12 h light period. Single spore isolates were obtained from a spread of conidial suspension on water agar. For this, a diluted spore suspension was transferred to water agar and incubated at 26°C for 16 h. Then a germinating and unbranched single spore was transferred to PDA for further analysis.

### Morphological characterization

**Figure 2:**
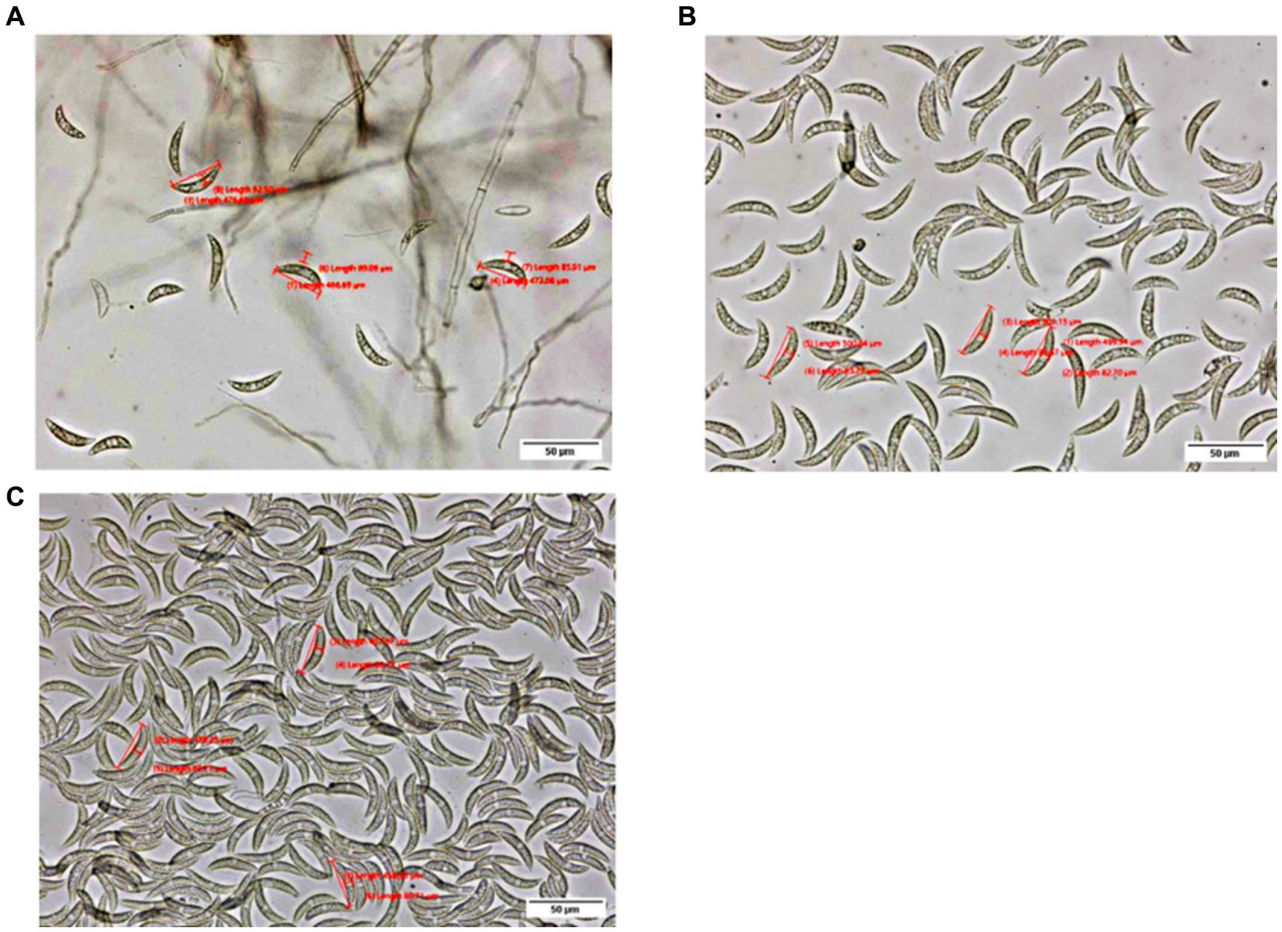
Pictures depict low **(A)**, medium **(B)**, and high **(C)** conidia populations, as observed under an OLYMPUS-SC50 digital compound microscope at a 40× objective lens.

A total of 114 pathogen isolates were morphologically characterized using an OLYMPUS-SC50 digital compound microscope. The morphological characterization of the isolates was conducted on the 7th day of incubation except for colony size which was measured for four consecutive days at a 24 h interval starting at 48 h of incubation. Data on conidia, appressoria, acervuli, and setae were recorded as described by Crouch et al. (2009). For morphological characterization, a sample was taken 1 cm from the center of the colony and smeared in a drop of water. The conidia populations were determined by counting the number of conidia in suspension under a compound microscope with a 40x objective lens and were categorized into four (3 = high, when the number of conidia was more than 300; 2 = medium, when the number of conidia was 100–300; 1 = low, when the number of conidia was 1–100; and 0 = none, when there were no conidia) (Figure 2). The conidia length and width were recorded from the mean of three conidia, while the maximum number of setae was counted. Among the 114 isolates, 22 isolates representing the four administrative zones (9, 3, 1, and 9 from Assosa, East Hararge, East Wollega, and Metekel, respectively) were selected based on geographical origin and morphological characters for virulence variability analysis. In addition, three morphologically uncharacterized isolates (two from Assosa and one from East Hararge) were added for the virulence analysis.

### Plant materials, inoculation, and disease assessment

#### Plant materials

In total, 25 sorghum genotypes were used for virulence analysis of the pathogen isolates and resistance analysis of the genotypes against the isolates at the Swedish University of Agricultural Sciences (SLU), Department of Plant Breeding, Alnarp, Sweden. A total of 18 genotypes were sorghum differentials provided by Professor Tesfaye D. Mengiste (Purdue University, Department of Botany and Plant Pathology, United States), while 7 genotypes (six landraces and one variety, Bonsa) were obtained from the Ethiopian Institute of Agricultural Research, MARC, Ethiopia. The sorghum genotypes SC748-5 and BT × 623 were included as resistant and susceptible checks, respectively. In addition to the improved variety Bonsa, two landrace genotypes (TSL_100090 and ETSL_100267) were regarded as resistant, while two landrace genotypes (ETSL_100039 and ETSL_101249) and two landrace genotypes (ETSL_101388 and ETSL_100761) were regarded as moderately resistant and susceptible, respectively, based on data obtained from field trials conducted at four locations in Ethiopia during the 2022 cropping season (data not shown).

#### Experimental design and inoculation

In this study, detached leaf assay was used as it has been reported to give comparable results to direct inoculation of the whole plant under greenhouse conditions (Prom et al., 2016). For this, sorghum genotypes were planted in 3.5 L volume plastic pots filled with potting mix at SLU’s greenhouse in Alnarp. Forty days after planting, the sixth leaves of each seedling were cut into 5–6 cm long segments and transferred to a 9 cm Petri dish containing double filter papers for detached leaf assay. A split-plot design, with 25 sorghum genotypes as main plots and 25 fungal isolates as subplots, was used. In each Petri dish, three leaf segments from each plant were used. To fix the leaf segment to the filter papers and prevent drying, 5 mL sterilized distilled water was added to the filter papers, and leaf margins were covered with sterile tissue paper. A hemacytometer was used to adjust the spore concentration from 8 to 10 days-old culture to 1 × 10^6^ spores/ml. Each leaf segment was inoculated at two locations with a drop of 30 μL conidia suspension. In total, 625 Petri dishes and 1875 leaf segments were used in this experiment.

### Disease assessment and data collection

A 1 to 5 scoring scale modified from (Cuevas et al., 2021) was used to assess the infection level, where 1 = no disease symptom; 2 = red spots on the inoculated area but no acervuli formation hypersensitive reaction (HR) (1–10%); 3 = red lesions on the inoculated area and few spots with acervuli but not expanding (11–25%); 4 = red lesions and visible expanded acervuli spots on the inoculated area (26–50%), and 5 = abundant acervuli covered lesions beyond the inoculated area with dead tissues in the middle (more than 50%). The infection progress was photographed with a fixed Canon digital camera at 60, 84, 108, 132, 156, and 180 h post-inoculation (hpi) followed by image analysis. The genotype’s reaction scores (pathogen infection level) 1 and 2 are categorized as resistant, while scores 3–5 are categorized as susceptible. Pathogen re-isolation was conducted from 20 randomly selected infected leaf segments to confirm that the observed infection was due to the inoculated pathogen. For this purpose, infected leaf segments were assessed following the same method described earlier for pathogen isolation.

### Determining hyphal growth of *Colletotrichum sublineola* isolates

Re-inoculation of the pathogen to new leaf segments was carried out to determine the structural growth of fungal hyphae and to evaluate the response of resistant and susceptible sorghum genotypes. For this, three pathogen isolates (AS106_F43a, NK73_F37, and PW123_F47) and three sorghum genotypes (BT × 623, SC748-5, and ETSL_1001249) were selected based on their reaction in the detached leaf assay. The analysis was conducted to determine the host–pathogen interaction and the structural growth of the fungal pathogen within the leaf. The assessment was conducted on the 7th day of inoculation. As a procedure, infected tissue was immersed in 100% ethanol for 2 days followed by 10% KOH for 2 h at 85°C to facilitate bleaching and complete chlorophyll removal. Subsequently, WGA-AF488 (green) was used to stain the fungal hyphae, while propidium iodide (red) was employed to stain dead host cells, providing a contrasting view as described in Redkar et al. (2018). The fungal hyphae and plant cell structure images were taken under a confocal microscope.

### Data analysis

The morphological and disease severity data were subjected to ANOVA using PROC GLM (SAS version 9.4). Euclidean distance-based cluster analysis was used to determine the clustering pattern of the pathogen isolates based on the morphological data in the R package “hclust” (Savje, 2024). The Elbow method was used to determine the optimal number of clusters. The Corrplot package in R software was used to determine the correlation between the morphological traits of the pathogen isolates. ImageJ software was used to take images under a confocal microscope (Redkar et al., 2018).

## Results

### Morphological diversity of *Colletotrichum sublineola* isolates

Statistical analysis revealed significant differences (*p* < 0.05) among the *C. Sublineola* isolates in their conidia length, conidia width, colony size, and number of setae (Table 2). All the isolates had falcate conidia except PW84_F37, which had a mixture of falcate and flat conidia. The longest conidia (26.25 μm) was observed in the isolate PW90_F40, followed by PW74_F34 (26.13 μm), while the shortest conidia were found in isolates AS110_F43 (16.93 μm) and PW84_F37 (17.78 μm) (Table 2). The widest conidia (5.3 μm) was recorded in an isolate from Assosa (AS105_F43) followed by two isolates from Pawe (PW83_F37 = 5.28 μm and PW122_F47 = 5.1 μm). After 7 days of incubation, the largest colony (6.82 cm) was exhibited by isolates NK73_F37 and PW83_F37, whereas the smallest colony (2.2 cm) was recorded for isolate PW84_F37. Colony color was varied in all the isolates ranging from dirty white to gray and dark green. In general, isolates from Assosa and Nekamte showed a gray colony color at the center and dirty white at the margin, whereas isolates from Pawe showed dark green, light green, and salmon gray. However, all three isolates from Haramaya showed a dark green colony color. The maximum number of setae were recorded at Assosa from four isolates (AS100_F43 = 42.33, AS108_F43 = 39, AS90_F40 = 32, AS111_F43 = 21.67) (Table 2).

**Table 2:** Morphological description and variation of 22 *C. sublineola* isolates characterized in this study.

Isolates	Conidia length (μm)	Conidia width (μm)	Colony size (cm)	Colony color	Number of setae
PW123_F47	21.3	5.0165	4.17	Salmon gray	20.33
PW117_F46	22.4835	4.3835	4.2	Salmon-gray	1.33
AS100_F43	23.4	4.3665	3.38	gray	42.33
PW74_F34	26.1335	4.6665	3.1	Dark green	1.33
PW122_F47	22.5835	5.1	3.72	Salmon-gray	3
AS110_F43	16.9335	4.6835	3.32	Light green	3
HU22_F04	24.4335	4.5335	4.03	Dark green	1.33
AS90_F40	22.9165	3.7665	3.77	gray	32
AS111_F43	23.3665	4.55	3.4	Light green	21.67
AS89_F40	23.55	4.2665	3.8	Light green	5
AS106_F43a	23.2835	4.4	3.32	gray	1.33
PW90_F40	26.25	4.1165	2.5	Light green	2.33
HU34_F06	22.8165	4.7835	3.67	Dark green	2.33
NK73_F37	21.2335	4.4665	6.82	Dirty white	14.33
HU14_F02	–	–	–	–	–
PW83_F37	19.1665	5.2835	6.82	Dark green	1.33
AS74_F33	–	–	–	–	–
PW84_F37	17.7835	4.4665	2.2	Dark green	1.67
AS105_F43	25.0835	5.3	3.55	Gray	14.67
PW85_F37	20.2665	4.65	4.97	Dark green	14.33
AS106_F43a	–	–	–	–	–
HU28_F06	NA	NA	4.93	Dark green	2.67
AS108_F43	22.5335	4.35	3.72	Gray	39
AS104_F43	24.9335	4.15	4.5	Gray	11.67
PW125_F47	22.0335	4.3335	2.93	White	8
Mean	22.5	4.55	3.95		11.14
LSD	1.71	0.64	0.34		1.61
CV	4.61	8.5	5.2		8.77
*p*-value	<0.0001	<0.0028	<0.0001		<0.0001

NA, not available; –, not morphologically characterized data.

### Clustering of *Colletotrichum sublineola* isolates

**Figure 3:**
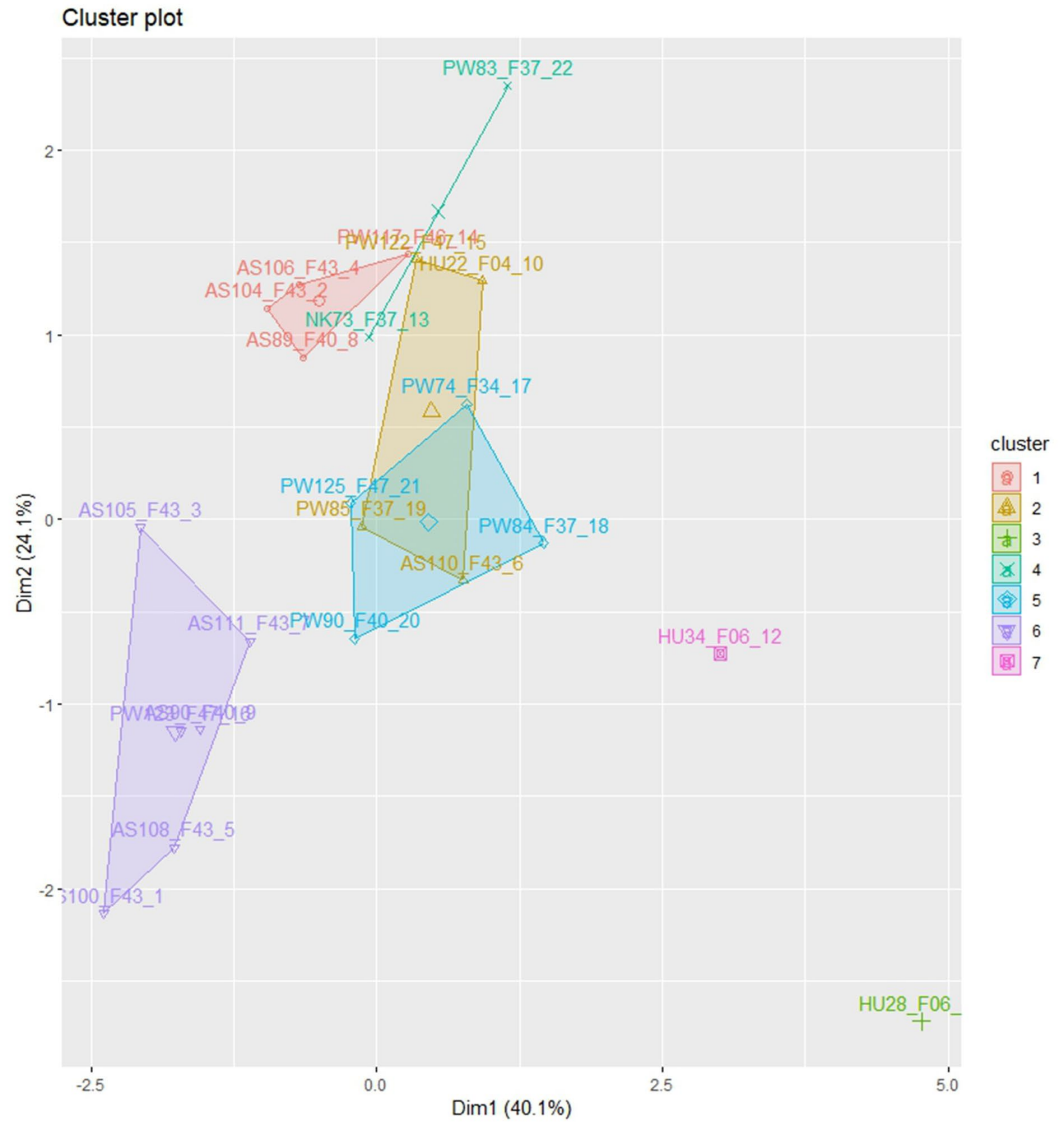
The clustering of 22 *C. sublineola* isolates in morphological variations, collected from four different administrative zones in Ethiopia.

Euclidian distance-based cluster analysis was employed to visualize the relationships between the isolates (Figure 3). The analysis categorized the isolates into seven distinct morphological groups with low levels of admixture. Isolates from Assosa were predominantly grouped in clusters I and VI, while isolates from Pawe were distributed across clusters I, II, IV, V, and VI. Cluster III consisted of isolate HU28_F06 obtained from Haramaya while isolating HU34_F06 from the same area was assigned to cluster VII. Cluster IV comprised isolates from Nekamte (NK73_F37) and Pawe (PW83_F37). Isolates from Pawe were distributed across five clusters (I, II, IV, V, and VI), indicating their morphological diversity (Figure 3). The clustering analysis demonstrated that the isolates showed significant morphological variation with some degree of association with their geographical origin.

### Correlation analysis

**Figure 4:**
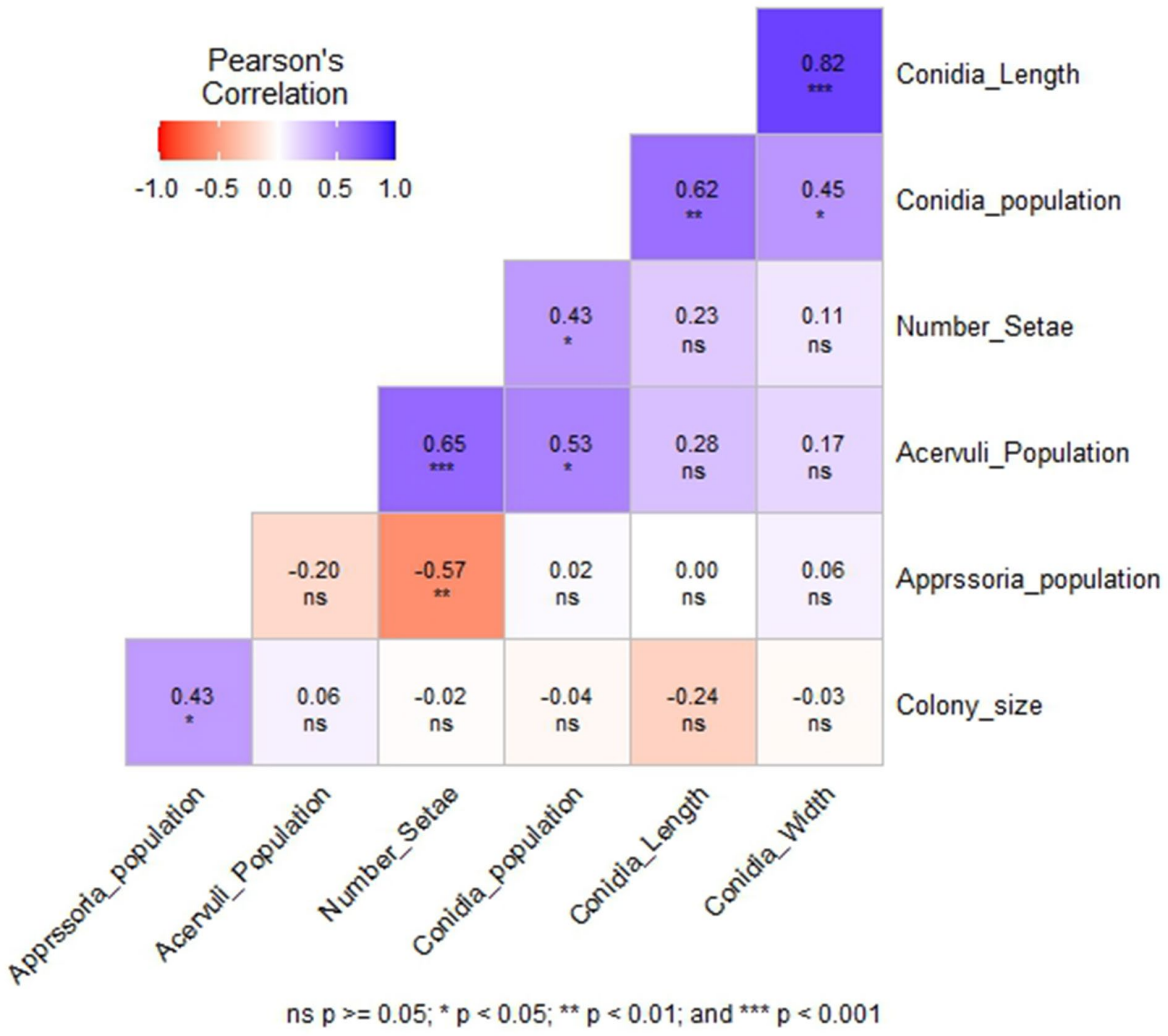
The correlation between six morphological variables of *C. sublineola* isolates collected from four administrative zones in Ethiopia.

Among the six morphological variables, the number of setae had a highly significant positive correlation with the acervuli population (*p* < 0.001) and a significant negative correlation with the appressoria population (*p* < 0.01) (Figure 4). Conidia width and length showed a highly significant positive correlation (*r* = 0.82, *p* < 0.001). However, they were not correlated with colony size, acervuli population, number of setae, and appressoria population. Acervuli population, number of setae, and conidia population showed significant positive correlations (*r* = 0.43–0.65) (Figure 4). In general, isolates with a high acervuli population had a high conidia population but were not correlated with the appressoria population.

### Virulence level of *Colletotrichum sublineola* isolates and reaction of sorghum genotypes

**Figure 5:**
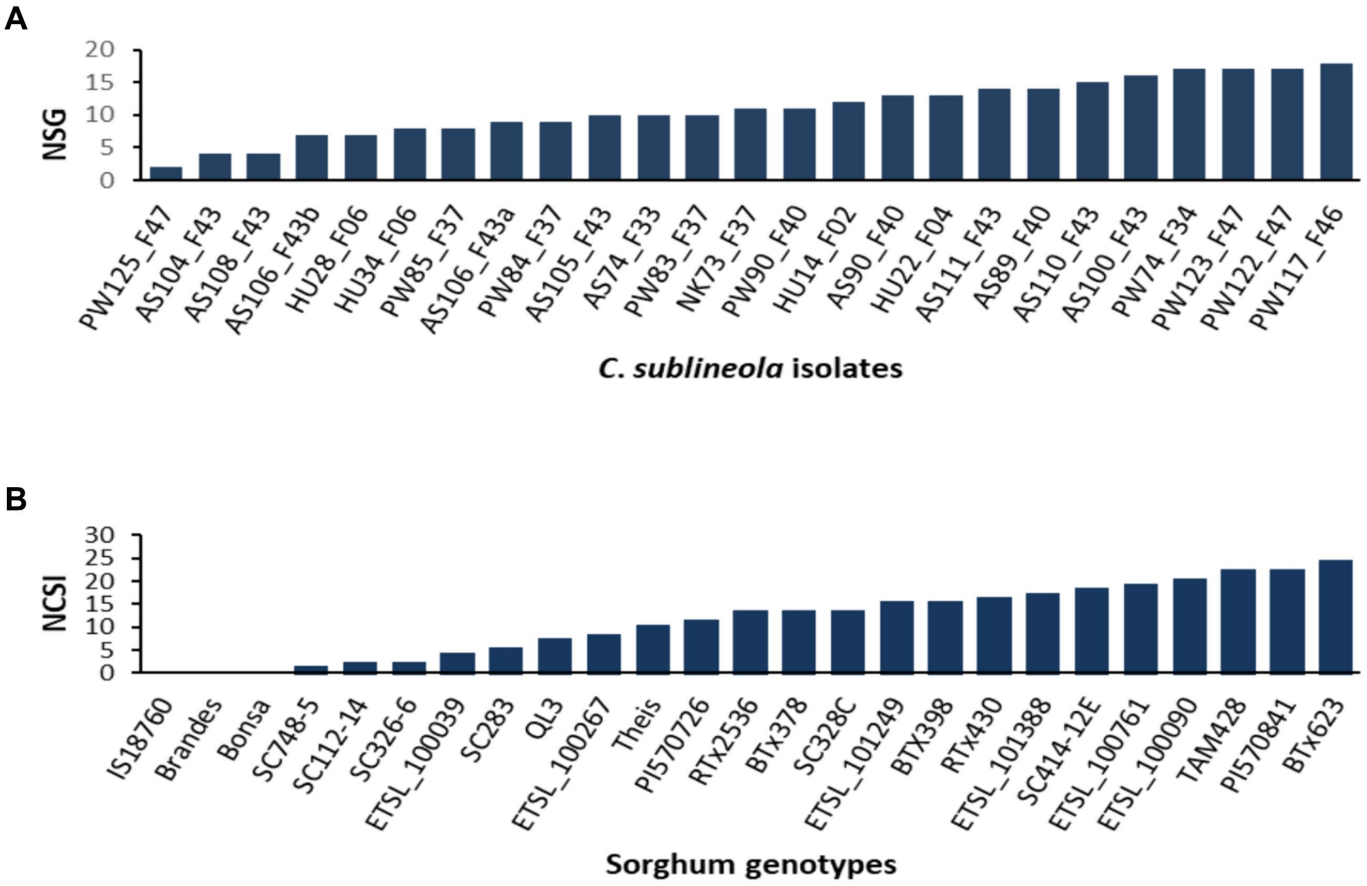
Bar graphs showing **(A)** the number of sorghum genotypes infected by each of the 25 *C. sublineola* isolates, and **(B)** the number of *C. sublineola* isolates that infected each of the 25 sorghum genotypes. NSG, number of sorghum genotypes; NCSI, number of *C*. *sublineola* isolates.

The analysis of variance (ANOVA) revealed significant differences among sorghum genotypes, *C. sublineola* isolates, and their interactions (Table 3). Among the 25 tested isolates, the top 4 most virulent pathotypes were from Pawe (PW122_F47, PW117_F46, PW74_F34, and PW123_F47) followed by pathotypes from Assosa (Figure 5). Pathotype PW117_F46, which infected 18 (72%) of the sorghum genotypes, was the most virulent followed by pathotypes PW74_F34, PW122_F47, and PW123_F47, which infected 17 (68%) sorghum genotypes. Pathotype AS100_F43 infected 16 (64%) genotypes, including BT × 623 (Figure 5), whereas pathotypes PW125_F47, AS108_F43, and AS104_F43 were the least virulent, which infected only two, four, and five of the tested sorghum genotypes, respectively (Figure 5). Two pathotypes (HU28_F06 and HU34_F06) from Haramaya infected 28 and 32% of the sorghum genotypes tested, and hence, they are categorized as less virulent. Pathotypes HU14_F02 and HU22_F04 infected 52% of the sorghum genotypes tested; hence, their virulence is intermediate. Similarly, pathotype NK73_F37 collected from Nekamte showed an intermediate virulence level, even though it infected the sorghum genotype SC748-5, which was widely regarded as resistant to anthracnose. In total, 8 (AS100_F43, AS104_F43, AS105_F43, AS106_F43a, AS106_F43b, AS108_F43, AS110_F43, and AS111_F43) of the 11 isolates from Assosa were collected from the same field, and interestingly they exhibited different virulence levels. In addition, isolates isolated from the same leaf sample (AS106_F37a and AS106_F37b) showed different virulence pattern and colony color.

**Table 3:** ANOVA table with their *F* and *p* values for disease severity of 25 *C. sublineola* isolates on 25 sorghum genotypes.

Source of variations	df	Sum of squares	Mean squares	*F* value	*p* value
Genotypes	24	178224.41	7426.02	496.80	<0.0001
Isolates	24	106971.66	4457.15	298.18	<0.0001
Genotypes*Isolates	576	270019.25	468.78	31.36	<0.0001

df, degree of freedoms.

The ANOVA revealed significant differences (*p* < 0.05) between the *C. sublineola* isolates in anthracnose severity (Table 4). Among the tested isolates, PW123_F47 and PW117_F46 were the most aggressive, causing 31% anthracnose severity. AS100_F43 and PW74_F34 were the second-most aggressive pathotypes with 28% anthracnose severity (Table 4). *C*. *sublineola* Pathotypes vary considerably in their virulence and aggressiveness. For instance, AS106_F43a and AS106_F43b, which were isolated from the same leaf sample, showed differences in their virulence. Pathotype AS106_F43a was more aggressive than AS106_F43b, with severity scores of 17.87 and 8.87%, respectively. Moreover, AS106_F43a, which infected nine genotypes, was a bit more virulent than AS106_F43b, which infected seven genotypes. Pathotype PW125_F47 from Pawe was less virulent as it only infected two genotypes, BT × 623 and ETSL_100090. It was also the least aggressive pathotype with a 5.87% severity score.

**Table 4:** Mean comparison of severity percentage (aggressiveness) of 25 *C. sublineola* isolates and severity level of 25 sorghum genotypes.

Isolates	Isolates severity (%)	Genotypes^a^^,^^b^	Genotype severity (%)
PW123_F47	31	PI57084^b^	35.8
PW117_F46	30.85	ETSL_101249^a^	33.71
AS100_F43	28.17	BT × 623^b^	32.28
PW74_F34	28.08	TAM428^b^	28.97
PW122_F47	27.8	ETSL_100090^a^	27.83
AS110_F43	23.23	SC414-14^b^	26.01
HU22_F04	20.09	RT × 430^b^	25.33
AS90_F40	18.99	SC328C^b^	23.52
AS111_F43	18.27	PI57072^b^	19.61
AS89_F40	17.97	ETSL_101388^a^	18.28
AS106_F43a	17.87	ETSL_100761^a^	17.84
PW90_F40	17.61	ETSL_100267^a^	17.67
HU34_F06	16.64	RT × 2536^b^	16.16
NK73_F37	16.2	BT × 398^b^	15.97
HU14_F02	14.95	Theis^b^	14.84
PW83_F37	14.27	BT × 378^b^	11.87
AS74_F33	13.11	QL3^b^	9.95
PW84_F37	12.39	SC748-5^b^	8.48
AS105_F43	11.89	SC112-14^b^	8.31
PW85_F37	9.32	SC283^b^	7.76
AS106_F43b	8.87	ETSL_100039^a^	6.24
HU28_F06	8.76	SC326-6^b^	5.57
AS108_F43	6.55	IS18760^b^	4.95
AS104_F43	6.19	Brandes^b^	4.49
PW125_F47	5.87	Bonsa^a^	3.48
**Mean**	**16.99**		**16.99**
**LSD**	**1.24**		**1.24**
**CV**	**22.74**		**22.74**
	*p* < 0.05		*p* < 0.05

a Genotypes from MARC (Ethiopia).

b Differential genotypes from Purdue university (USA).Mean: mean disease severity by 25 isolates and the mean disease severity on 25 genotypes.LSD: the list significant difference between treatments and any differences larger than this value are significant.CV: coefficient of variation showing level of variability of disease severity among isolates and genotypes.

Generally, Ethiopian sorghum genotypes showed inconsistent reactions, except for the variety Bonsa, which is regarded as universally resistant, and genotype ETSL_100761, which was susceptible to 19 of the 25 isolates. Genotype PI57084 was found to be the most susceptible (disease severity = 35.8%) followed by the genotypes ETSL_101249 and BT × 623, which had severity scores of 33.71 and 32.28%, respectively (Table 4).

### Virulence pattern and pathotype determination

**Figure 6:**
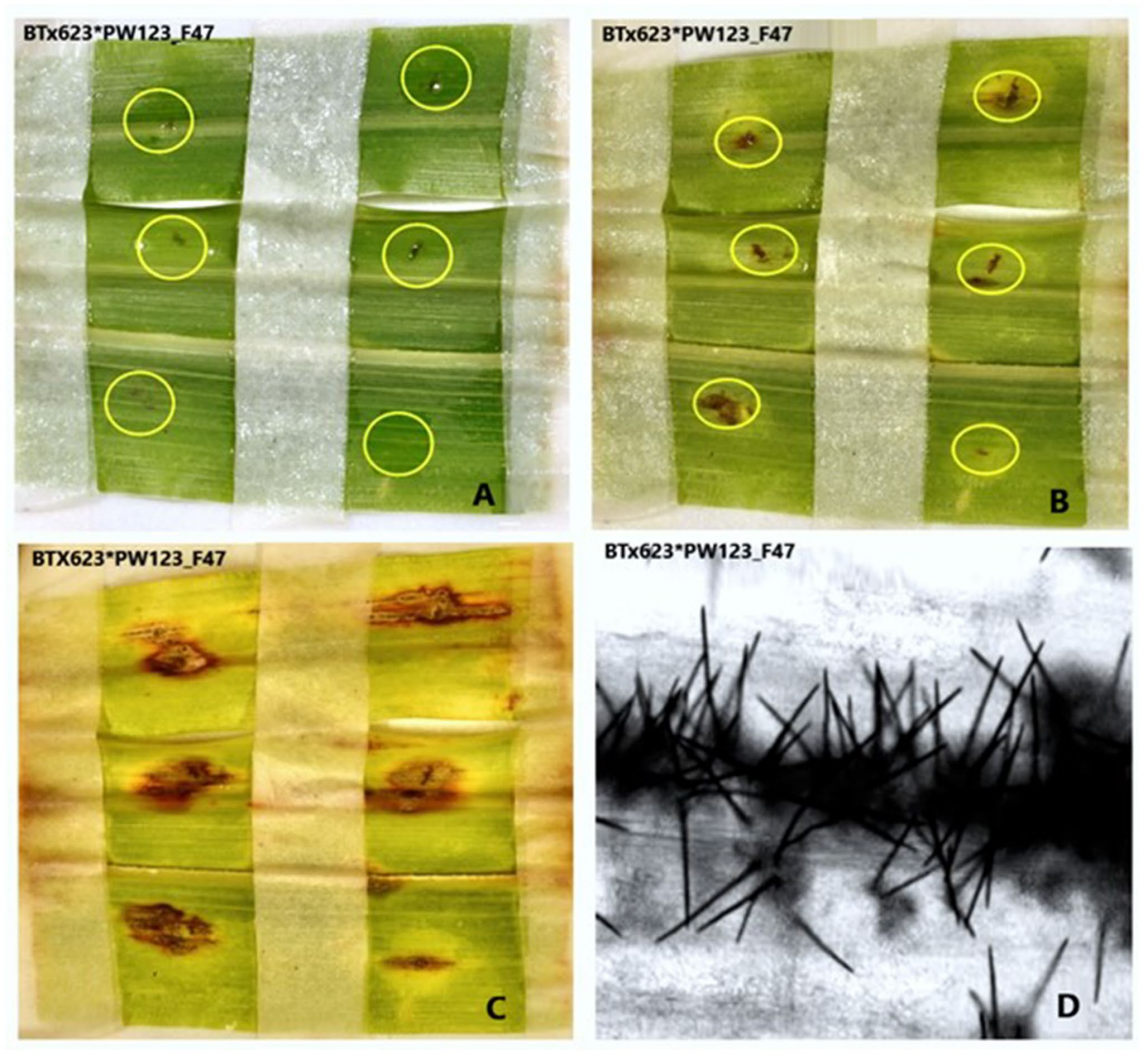
Pictures depicting the progress of anthracnose caused by *C. sublineola* isolate PW123_F47 to sorghum genotype BT × 623. **(A)** Second dpi, **(B)** fifth dpi, **(C)** eighth dpi, and **(D)** the development of Setae on the eighth day post inoculation (dpi) as observed under a confocal microscope.

The resistance screening revealed significantly different resistance levels among the tested sorghum genotypes. None of the *C. sublineola* isolates infected the sorghum genotypes IS_18760, Brandes, and Bonsa (Table 5). Conversely, genotype BT × 623 was susceptible to all isolates (Figure 6), except AS104_F43. Sorghum genotypes PI 570841 and TAM428 were susceptible to 22 (88%) of tested isolates. Genotype SC748-5 was susceptible to pathotype NK73_F37, while genotype SC112-14 was susceptible to pathotypes PW123_F47 and PW122_F47 (Table 5). Both SC748-5 and SC112-14 genotypes were previously reported as resistant genotypes (Prom et al., 2023). Among the Ethiopian genotypes, the variety Bonsa was the most resistant as it was not infected with any of the pathotypes. ETSL_100039 and ETSL_100267 were infected by four and eight pathotypes, respectively. ETSL_100090 was not infected and showed anthracnose symptoms under field conditions. However, it was susceptible to 80% of the pathotypes under greenhouse conditions and was the most susceptible among the Ethiopian sorghum genotypes. Genotype ETSL_100761 showed a susceptible reaction to most of the isolates in line with the field trial data. In general, the virulence pattern of 25 *C*. *sublineola* isolates tested against 25 sorghum genotypes revealed 24 pathotypes (Table 5).

**Table 5:** Pathotype determination and virulence pattern of 25 *C. sublineola isolates* on 25 sorghum genotypes.

Genotypes	Isolates																									
	AS100_F43	AS104_F43	AS105_F43	AS106_F43a	AS108_F43	AS110_F43	AS111_F43	AS74_F33	AS89_f40	AS90_F40	AS106_F43b	HU14_F02	HU22_F04	HU28_F06	HU34_F06	NK73_F37	PW117_F46	PW122_F47	PW123_F47	PW74_F34	PW83_F37	PW84_F37	PW85_F37	PW90_F40	PW125_F47	NGSR
IS18760	R	R	R	R	R	R	R	R	R	R	R	R	R	R	R	R	R	R	R	R	R	R	R	R	R	0
Brandes	R	R	R	R	R	R	R	R	R	R	R	R	R	R	R	R	R	R	R	R	R	R	R	R	R	0
Bonsa	R	R	R	R	R	R	R	R	R	R	R	R	R	R	R	R	R	R	R	R	R	R	R	R	R	0
SC748-5	R	R	R	R	R	R	R	R	R	R	R	R	R	R	R	S	R	R	R	R	R	R	R	R	R	1
SC112-14	R	R	R	R	R	R	R	R	R	R	R	R	R	R	R	R	R	S	S	R	R	R	R	R	R	2
SC326-6	R	R	R	R	R	R	R	R	S	R	R	R	R	R	R	R	R	R	R	S	R	R	R	R	R	2
ETSL_100039	S	R	S	R	R	S	R	R	R	R	R	R	R	R	R	R	S	R	R	R	R	R	R	R	R	4
SC283	S	R	R	R	S	R	R	R	R	S	R	R	R	R	R	R	S	R	R	S	R	R	R	R	R	5
QL3	S	R	R	R	R	R	R	R	R	S	R	S	S	R	R	R	R	R	R	S	S	R	S	R	R	7
ETSL_100267	R	R	R	R	R	S	S	S	R	S	R	R	R	R	R	R	S	S	S	S	R	R	R	R	R	8
Theis	R	R	R	S	R	S	R	R	S	R	R	R	R	S	S	R	S	S	S	R	S	R	R	S	R	10
PI570726	S	R	R	R	R	S	S	R	R	R	R	R	R	R	S	S	S	S	S	S	R	S	R	S	R	11
RT × 2,536	R	R	S	R	R	S	S	R	S	R	S	S	S	R	R	R	S	S	S	S	S	R	R	S	R	13
BT × 378	S	R	R	R	S	S	R	S	S	S	R	S	S	R	R	S	S	S	S	S	R	R	R	R	R	13
SC328C	S	R	R	S	R	S	S	S	S	R	R	R	R	R	S	S	S	S	S	R	R	S	S	R	R	13
ETSL_101249	S	R	R	S	R	S	S	R	S	S	R	S	S	R	S	R	S	S	S	S	R	S	R	S	R	15
BT × 398	S	R	S	R	R	R	S	R	S	S	S	S	S	R	R	S	S	S	S	S	S	R	S	R	R	15
RT × 430	S	R	S	R	R	S	S	R	S	S	R	S	S	R	R	S	S	S	S	S	S	S	R	S	R	16
ETSL_101388	S	S	S	S	R	S	S	S	S	R	R	S	S	S	R	R	S	S	S	S	R	S	R	S	R	17
SC414-112E	S	R	R	R	R	S	S	S	S	S	S	S	S	R	S	S	S	S	S	S	S	S	R	S	R	18
ETSL_100761	S	S	S	S	S	S	S	S	R	S	S	S	S	S	R	S	S	S	S	S	R	R	S	R	R	19
ETSL_100090	S	S	S	S	R	R	S	S	S	S	R	S	S	S	R	R	S	S	S	S	S	S	S	S	S	20
TAM428	S	R	S	S	R	S	S	S	S	S	S	S	S	S	S	S	S	S	S	S	S	S	S	S	R	22
PI570841	S	S	S	S	R	S	S	S	S	S	S	S	S	S	S	S	S	S	S	S	S	R	S	S	R	22
BT × 623	S	R	S	S	S	S	S	S	S	S	S	S	S	S	S	S	S	S	S	S	S	S	S	S	S	24

NGSR, number of genotypes with susceptible reactions.

### Re-inoculation and evaluation of fungal hyphae growth

**Figure 7:**
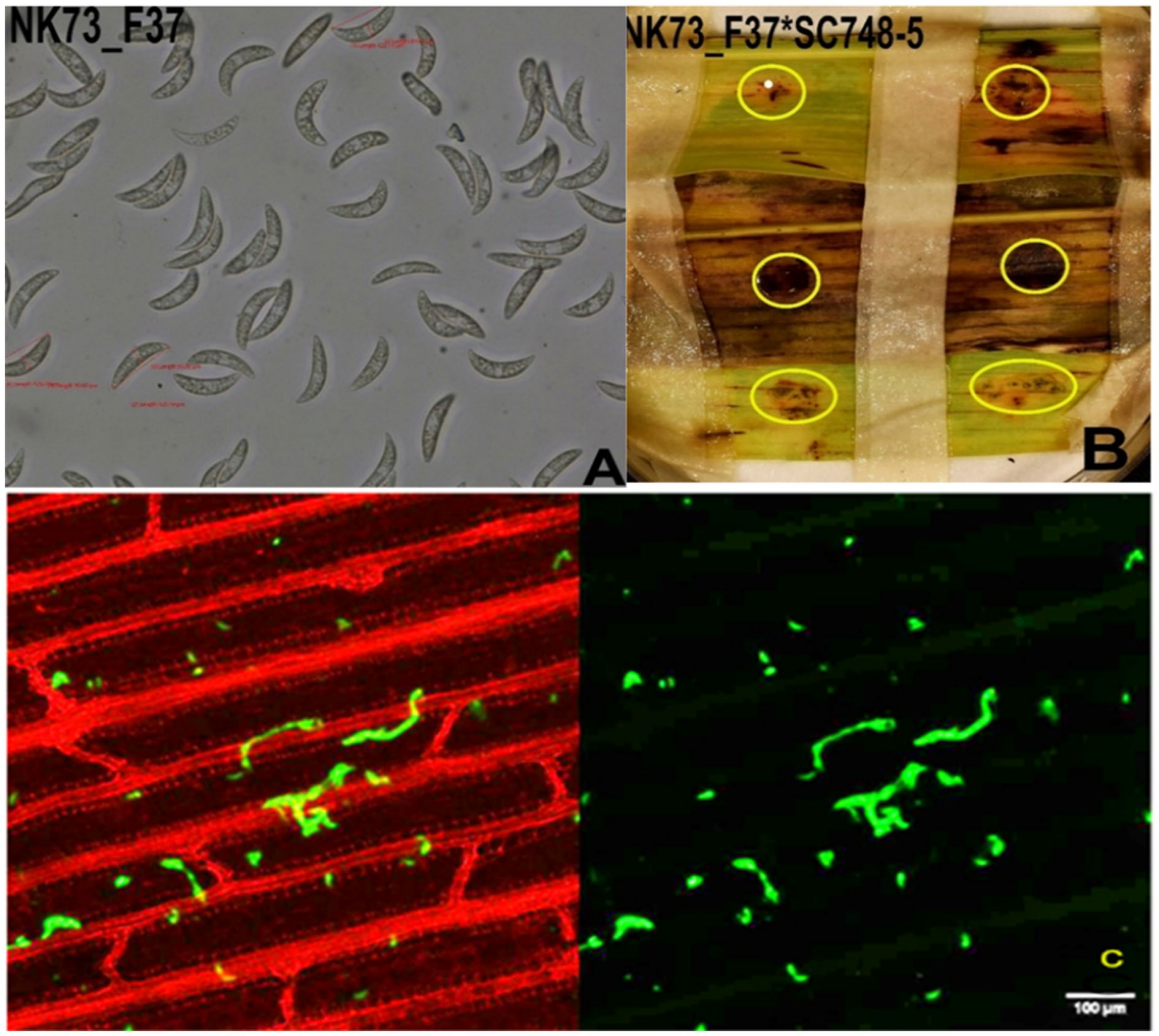
Infection of sorghum genotype SC748-5 by isolate NK73_F37. **(A)** Fungal conidia of NK73_F37; **(B)** Infection symptom development (acervuli circled with yellow rings), and **(C)** the growth of fungal hyphae of NK73_F37 in the leaf tissue of BT × 623 on the 7th dpi observed under a confocal microscope. Scale bars = 100 μm.

Figure 7 presents the reaction of SC748-5 sorghum genotype against NK73_F37 isolate. The symptom development was similar to the previous artificial inoculation for all three sorghum genotypes (BT × 623, SC748-5, and ETSL_1001249) to three *C. sublineola* isolates (AS106_F43a, NK73_F37, and PW123_F47) recorded on the seventh day of post inoculation (dpi). However, the hyphal growth was not clearly visible in genotype SC748-5 under a confocal microscope as in BT × 623 and ETSL_101249 (Figure 7C). Furthermore, the infected spots were not expanded to create coalescence in nearby spots. Such limited growth of symptoms could be an indication of a late response of SC748-5 to the inoculated isolate NK73_F37.

## Discussion

Anthracnose of sorghum, caused by the fungus *C. sublineola*, poses a significant threat, leading to substantial losses in grain and biomass yields. The pathogen exhibits remarkable variation in symptoms, morphology, and pathogenicity, influenced by changes in environmental conditions and host factors (Valério et al., 2005; Chala, 2013). Understanding this variation within the pathogen is critical for developing durable and effective management strategies. This study employed morphological and virulence assessments to elucidate the interaction between *C. sublineola* isolates and sorghum genotypes. The methods employed in this study, constituting part of a polyphasic approach, facilitated the delineation of isolate variation. By utilizing a variety of sorghum anthracnose differentials, both qualitative (virulence) and quantitative (aggressiveness) distinctions among isolates were elucidated. The level of virulence provided insights into the presence of potential a virulence genes within the isolates and resistant genes within the host, contributing to a better understanding of the pathogen–host interaction.

The study revealed significant morphological and virulence variation among isolates, including between isolates recovered from the same leaf. Variation in virulence could be found within a *C. sublineola* population (Erpelding, 2011) in addition to the morphological variation, which could help to understand variation among isolates of the same species (Cannon et al., 2012). In Assosa, 8 of the 11 isolates were collected from the same research field and exhibited variation in virulence. In addition, pathotypes AS106_F37a and AS106_F37b were isolated from the same leaf sample and differed in virulence and colony morphology. Differentiation between isolates collected from a single research station elucidated the high variability of the pathogen, implying a lack of random mating in the pathogen population within a field (Prom et al., 2012). Similarly, high diversity among 22 isolates collected from a single field in Ethiopia has been reported (Chala, 2013), which is In line with the current study.

ANOVA revealed significant differences among the tested *C. sublineola* isolates collected from major sorghum growing areas in Ethiopia. Variation among isolates may arise from geographical isolation and specialized adaptation to hosts. In line with this, Erpelding and Prom (2009) stated that *C. sublineola* has a global distribution, with diverse pathotypes observed across various climatic regions. The present study also revealed a wide distribution of *C. sublineola* in Ethiopia as well as considerable variations in their virulence pattern and morphological characteristics. The differences in pathogenicity and virulence of *C. sublineola* in the country imply the need for continuous assessment of the pathogen genetic variation. As part of this approach, resistance genes could be identified and used to manage sorghum anthracnose effectively and sustainably.

The presence of 24 pathotypes in 25 isolates including those collected from the same field and leaf that showed different virulence levels in sorghum differentials indicates extreme pathogen variability. Cluster analysis revealed that isolates in five of the seven clusters were grouped according to their geographical origin, suggesting a significant contribution of collection sites to isolate differences. However, isolate grouping in terms of colony morphology did not correlate with virulence level. For instance, the most virulent pathotype PW74_F34 was grouped with the non-virulent pathotype PW125_F43 in cluster V, and the virulent pathotype PW117_F43 was grouped with the non-virulent pathotype AS104_F46 in cluster I. Pathotypes from Pawe, including the four top-ranked virulent pathotypes were distributed across five clusters, indicating high pathotype diversity in this part of the country. Cluster IV contained one pathotype from Pawe (PW83_F37) and one pathotype from Nekamte (NK73_F37), both showing similar pathogenicity and virulence against the genotypes and having similar morphological characters except the lower number of setae in pathotype PW83_F37. This may suggest a wide geographical distribution of some *C. sublineola* variants.

Although the infection of the sorghum genotype SC748-5 by pathotype NK73_F37 may suggest the emergence of a new virulent *C. sublineola* variant in Ethiopia, further study should be conducted for deeper insight including the gene-for-gene interaction. In the genus *Colletotrichum,* the emergence of genetic variants is common due to heterokaryosis, sexual cycle, transposable elements, and point mutation (Lopes et al., 2020). While the significance of the sexual cycle in generating genetic diversity is obvious and the role of heterokaryosis in the emergence of field variants remains uncertain, genetic differentiation of *C. sublineola* can result in resistance breakdown (Lopes et al., 2020). Isolation by distance and host diversification in the same field could contribute to the emergence of new pathogen variants. In sorghum anthracnose, many races have evolved to overcome LRR type R genes, and the host contains a large family comprising evolving LRR genes (Ahn et al., 2019). The present study revealed that the most virulent *C. sublineola* Pathotypes were collected from Pawe; this could be attributed to its favorable environment for the pathogen and a genetically diverse host cultivated in the area, which drives the pathogen toward increased pathogenicity and virulence compared to other areas. Similar observations were made regarding *Puccinia* species, where efforts to breed resistance in cereal crops introduced new selective pressures, resulting in the proliferation of virulent gene frequencies (Simon et al., 2020).

The tested sorghum genotypes showed different levels of resistance to the inoculated *C. sublineola* isolates ranging from no infection to being infected by multiple isolates. The sorghum genotypes IS_18760, Brandes, and Bonsa showed the highest resistance and were least affected by all 25 isolates, implying their broad-spectrum resistance to the *C. sublineola* variant. Hence, they could be a promising source of resistance genes for breeding sorghum cultivars resistant to anthracnose. Genotypes SC748-5 and SC112-14 were regarded as resistant to *C. sublineola* isolates, and genes on chromosome 5 contributed to their resistance (Valério et al., 2005; Prom et al., 2012; Prom et al., 2023). However, in the present study, genotype SC748-5 was susceptible to pathotype NK73_F37, and genotype SC112-14 was also susceptible to PW122_F47 and PW123_F47, indicating the presence of resistance-breaking *C. sublineola* pathotypes in Ethiopia. Conversely, sorghum genotype PI_57084 was the most susceptible followed by ETSL_101249, BT × 623, and TAM428. The present study also showed significant differences in aggressiveness among the tested *C. sublineola* isolates. Among the tested isolates, pathotypes PW123_F47 and PW117_F46 were the most aggressive causing anthracnose severity of 31%. This indicates that these isolates are useful for further sorghum germplasm screening for anthracnose resistance. Moreover, it indicates that Pawe is a hot spot for pathogen virulence and genetic variability analyses as well as for field evaluation of sorghum genetic resources for their response to *C. sublineola* infection.

The re-inoculation experiment involving known resistant and susceptible genotypes (SC748-5, BT × 623, and ETSL_1001249) with three isolates (NK73_F37, PW123_F47, and AS106_F43a) showed reproducibility of observed isolate–genotype interactions. However, proper structural growth of the fungi was observed only in genotypes BT × 623 and ETSL_1001249 (not SC748-5). Despite the fact that numerous anthracnose resistance loci have been reported in sorghum in recent years, resistance genes appeared to be not equally effective in different sorghum growing regions because of diverse *C*. *sublineola* pathotypes (Stutts and Vermerris, 2020). The limited fungal structural growth in SC748-5 could be because it possesses a dominant transcription factor gene conferring anthracnose resistance (Prom et al., 2012), and regulates gene expression patterns (Buiate et al., 2017). In general, a hypersensitivity reaction develops from plant-gene-producing pattern recognition receptors (PRRs) (Abreha et al., 2021), which recognize specific molecular patterns associated with pathogens (Zhang et al., 2014; Couto and Zipfel, 2016).

The infection symptom observed in the known resistant genotype (SC748-5) could be (1) late recognition of the pathogen by the host, which could lead to late response (Künstler et al., 2016), (2) the inoculated isolate NK73_F37 had evolved effectors that overcame the host resistance genes (Möller and Stukenbrock, 2017), which led to a change in the mechanism of infection, or (3) the pathogen might have initially colonized the host and remained in a latent or quiescent state for an extended period until it begins to macerate a plant tissue (Cannon et al., 2012) or combinations of these. In all cases, low or delayed host defense-related gene expression could occur. The dynamic nature of *C*. *sublineola* pathotypes, and resistant encoding genes associated with environmental factors could influence the host reaction time.

Cluster analysis conducted to visualize the relationships among the isolates revealed seven groups with some levels of admixture. None of the clusters exclusively contained isolates of a single geographical area indicating gene flow between isolates in different areas through various means like infected seed exchange and a step-by-step distribution of fungal spores over long distances. In line with this, Mathur et al. (2003) and Marley et al. (2005) stated that *C. sublineola* isolates obtained from different locations, the morphology of isolates extracted from a single-lesion culture, and even single-conidial derivatives of a single-lesion culture can vary considerably. Grouping the isolates based on their infection pattern and virulence level resulted in 24 pathotypes. Similar to the present finding, approximately 40 anthracnose pathotypes were identified among geographically diverse isolates tested under greenhouse and field conditions (Smith and Frederiksen, 2000; Mathur et al., 2003; Zanette et al., 2009). In Brazil, 22 pathotypes were identified among 37 isolates using 10 differentials (Valério et al., 2005). In the United States, Prom et al. (2012) reported 17 pathotypes among 18 isolates using 18 sorghum differentials, while Prom et al. (2023) reported 27 new pathotypes among 30 isolates using the same differential lines. This indicates that *C. sublineola* is a highly variable fungal pathogen.

## Conclusion

This study revealed that *C*. *sublineola* isolates from major sorghum growing areas in Ethiopia were diverse in virulence patterns and aggressiveness. Variation in virulence was observed even between isolates extracted from plants in the same field in a single sample, indicating the pathogen’s extreme variation. *C. sublineola* isolates collected from Pawe were highly virulent and aggressive, indicating that the area is suitable for studies on pathogen diversity and host-resistance screening against anthracnose. For instance, four isolates (PW122_F47, PW117_F46, PW74_F34, and PW123_F47) collected from Pawe were the most virulent (on 68–72% of sorghum genotypes). Among the 25 tested sorghum genotypes, three genotypes (IS_18760, Brandes, and Bonsa) were resistant to all tested isolates, hence, they could serve as potential sources of resistance genes to be utilized in sorghum breeding programs aimed at developing sorghum cultivars resistant to a variety of *C. sublineola* pathotypes. On the other hand, some sorghum genotypes previously considered resistant to anthracnose, such as SC748-5 and SC112-14, were susceptible to some isolates. This suggests the need for continuous monitoring and further improvement of resistant germplasm through breeding. Overall, the present study confirmed the presence of a large number of *C. sublineola* pathotypes in Ethiopia which significantly differ in virulence. This study also documented the host resistance levels against the tested isolates. Sorghum genotypes resistant to various *C. sublineola* isolates were identified, so they can serve as sources of genes for developing cultivars to anthracnose through breeding. The current study only targeted areas highly prone to sorghum anthracnose. Thus, future studies should cover a broader geographic area where sorghum is grown in order to gain a more comprehensive understanding of the geographical distribution of *C. sublineola* pathotypes. Additionally, DNA marker-based genetic diversity analysis of *C. sublineola* populations in Ethiopia, as well as functional analysis of host-pathogen interactions, are highly recommended for effective control of sorghum anthracnose.

## Data Availability

The original contributions presented in the study are included in the article/supplementary material, further inquiries can be directed to the corresponding author.
